# Rapid and reliable identification of Gram-negative bacteria and Gram-positive cocci by deposition of bacteria harvested from blood cultures onto the MALDI-TOF plate

**DOI:** 10.1186/s12866-015-0459-8

**Published:** 2015-06-18

**Authors:** Simona Barnini, Emilia Ghelardi, Veronica Brucculeri, Paola Morici, Antonella Lupetti

**Affiliations:** Dipartimento di Ricerca Traslazionale e delle Nuove Tecnologie in Medicina e Chirurgia, Università di Pisa, Via San Zeno 35-39, 56127 Pisa, Italy

**Keywords:** Blood culture, Bacteria, Direct identification, Bactec FX, MALDI-TOF, Vitek 2

## Abstract

**Background:**

Rapid identification of the causative agent(s) of bloodstream infections using the matrix-assisted laser desorption/ionization time-of-flight (MALDI-TOF) methodology can lead to increased empirical antimicrobial therapy appropriateness. Herein, we aimed at establishing an easier and simpler method, further referred to as the direct method, using bacteria harvested by serum separator tubes from positive blood cultures and placed onto the polished steel target plate for rapid identification by MALDI-TOF. The results by the direct method were compared with those obtained by MALDI-TOF on bacteria isolated on solid media.

**Results:**

Identification of Gram-negative bacilli was 100 % concordant using the direct method or MALDI-TOF on isolated bacteria (96 % with score > 2.0). These two methods were 90 % concordant on Gram-positive cocci (32 % with score > 2.0). Identification by the SepsiTyper method of Gram-positive cocci gave concordant results with MALDI-TOF on isolated bacteria in 87 % of cases (37 % with score > 2.0).

**Conclusions:**

The direct method herein developed allows rapid identification (within 30 min) of Gram-negative bacteria and Gram-positive cocci from positive blood cultures and can be used to rapidly report reliable and accurate results, without requiring skilled personnel or the use of expensive kits.

## Background

Blood culture is the gold standard to diagnose the causative agent(s) of bloodstream infections. To shorten the identification process, wide efforts have been made, including concentration of bacteria by centrifugation before direct inoculation of blood culture fluids into automated systems [1–4], and fluorescent in situ hybridization [5, 6]. In addition, several DNA-based techniques [7, 8], introduced with the aim to replace blood-culture systems technology, resulted to be useful as a complement, but not as a replacement of current automated systems [9, 10].

With the development of the matrix-assisted laser desorption/ionization time of flight (MALDI-TOF) mass spectrometry, the time required for accurate identification of microorganisms isolated on solid media to the species level reduced to a few minutes [11–13]. However, this method requires that bacteria from positive blood cultures are isolated on solid media, thus delaying bacterial identification by 24 h. The MALDI-TOF technology has the potential to be adapted to identify microorganisms grown in biological fluids [14]. To assess the use of MALDI-TOF for the identification of bacteria recovered from blood cultures, a variety of protocols (series of washes, centrifugations), protein extraction methods and analysis have been proposed [12, 15–22]. However, an easier method reducing the number of steps before the steel target plate of MALDI-TOF is prepared could be a real advantage for many clinical microbiology laboratories. In this study, we aimed at establishing a simple, reliable, and accurate method, further referred to as the direct method, using bacteria harvested from blood cultures by serum separator tubes (SST) and placed onto the polished steel target plate for identification by MALDI-TOF, in order to completely integrate the rapid method into the diagnostic routine. At first, the direct method was applied to blood cultures containing Gram-negative bacteria. The results obtained were compared with those by MALDI-TOF and Vitek 2 yielded with bacteria isolated on solid media the day after, further referred to as routine methods. When satisfactory results were obtained with Gram-negative bacteria, we applied the direct method to monomicrobial blood cultures containing Gram-positive cocci. In order to obtain favourable results with Gram-positive cocci, a protein extraction step performed on bacteria placed onto the polished steel target plate of MALDI-TOF resulted to be essential.

## Results

### Identification of Gram-negative bacteria from positive blood cultures

Positive blood cultures containing Gram-negative bacteria, that appeared monomicrobial at the Gram-stain, were selected for the identification of bacteria by the direct method with MALDI-TOF. Next, blood cultures were subcultured onto solid media and identified by routine methods. The results obtained by the direct and routine methods were compared.

Among the 133 positive blood cultures, 118 were monomicrobial after subculture. Direct identification of Gram-negative bacteria by MALDI-TOF gave an interpretable result for 117 (99.2 %) of 118 blood cultures (Table 1) with the following scores: 93 (78.9 %) score ≥ 2.3, 20 (17 %) score between 2.3 and 2.0, 3 (2.5 %) score between 2.0 and 1.7, and 1 (0.8 %) score < 1.7. Overall, 113 (95.9 %) of 118 blood cultures showed scores > 2.0. To assess the reproducibility of the identification, the bacteria were spotted in duplicate and the results revealed 100 % concordance and similar scores. MALDI-TOF identifications of bacteria isolated by the direct method or grown on solid media were always concordant (Table 1). A *Stenotrophomonas maltophilia* strain, which was not identified by the direct and routine methods with MALDI-TOF, was correctly identified by Vitek 2, as confirmed by additional biochemical tests.

**Table 1:** Identification of Gram-negative bacilli from monomicrobial positive blood cultures by MALDI-TOF (direct method)*

	No. of isolates						
Species	Identification by use of direct method MALDI-TOF MS with score (S) of:				No. of unidentified isolates		Total (%)
	S < 1.7	1.7 ≤ S < 2	2.0 ≥ S <2.3	S ≥ 2.3	Direct MALDI-TOF MS	Routine MALDI-TOF MS	
*Klebsiella pneumoniae*			4	40			44 (37.3 %)
*Escherichia coli*				23			23 (19.5 %)
*Enterobacter aerogenes*			1	4			5 (4.3 %)
*Enterobacter cloacae*			1	4			5 (4.3 %)
*Enterobacter cancerogenus*				1			1 (0.8 %)
*Klebsiella oxytoca*				3			3 (2.6 %)
*Proteus mirabilis*				3			3 (2.6 %)
*Serratia marcescens*			1	1			2 (1.7 %)
*Citrobacter freundii*				1			1 (0.8 %)
*Pseudomonas aeruginosa*		3	1	9			13 (11 %)
*Acinetobacter baumannii*			10	2			12 (10.2 %)
*Acinetobacter lwoffii*			1	1			2 (1.7 %)
*Pseudomonas stutzeri*			1				1 (0.8 %)
*Stenotrophomonas maltophilia*					1	1	1 (0.8 %)
*Bacteroides fragilis*				1			1 (0.8 %)
*Fusobacterium nucleatum*	1						1 (0.8 %)
Total (%)	1 (0.8 %)	3 (2.5 %)	20 (17 %)	93 (78.9 %)	1 (0.8 %)		118

*Using the results obtained with the routine methods by MALDI-TOF and Vitek 2 as comparators

No isolates were misidentified. No isolates were unidentified by Vitek 2

Among the 15 positive blood cultures that resulted to be polymicrobial after subculture (Table 2), one microorganism of the mixture was correctly identified to the species level by the direct method followed by MALDI-TOF in 13 (86.6 %) samples. Duplicates were 100 % concordant and gave similar scores. The scores achieved for 11 (73.3 %) of these samples were ≥ 2.0. In 2 polymicrobial samples, all microorganisms were unidentified. The mean identification time with the direct method was 20 min after blood culture positivity.

**Table 2:** Identification by MALDI-TOF (direct method) of Gram-negative bacilli from positive blood cultures appearing monomicrobial at the Gram-stain, that resulted to be polymicrobial after subculture*

Species	Identification by use of direct method MALDI- TOF MS				Total samples
	Correct, with score (S) of:			Unidentified species	
	1.7 ≤ S < 2	2.0 ≥ S < .3	S ≥ 2.3		
*Klebsiella pneumoniae*			1	*Escherichia coli*	1
*Klebsiella pneumoniae*		1		*Proteus mirabilis*	1
*Klebsiella pneumoniae*			2	*Acinetobacter baumannii*	2
*Klebsiella pneumoniae*		1		*Acinetobacter calcoaceticus*	1
*Klebsiella pneumoniae*	1			*Serratia marcescens*	1
*Escherichia coli*		1		*Pseudomonas aeruginosa*	1
*Enterobacter aerogenes*		1		*Candida guillermondii*	1
*Pseudomonas aeruginosa*		1		*Klebsiella pneumoniae*	1
*Pseudomonas aeruginosa*		1		*Candida tropicalis*	1
*Acinetobacter baumannii*		1		*Klebsiella pneumoniae*	1
*Acinetobacter baumannii*	1			*Staphylococcus epidermidis*	1
*Stenotrophomonas maltophilia*		1		*Candida glabrata*	1
No identification				*Lactobacillus paracasei* and *Candida krusei*	1
No identification				*Serratia marcescens* and *Klebsiella oxytoca*	1
Total (%)	2 (13.3)	8 (53.3)	3 (20 %)		15

*Using the results obtained with the routine methods by MALDI-TOF and Vitek 2 as comparators. No isolates were misidentified. No isolates were identified with scores ≤ 1.7

### Identification of Gram-positive cocci from positive blood cultures

Blood cultures, apparently monomicrobial for Gram-positive bacteria, were all containing Gram-positive cocci. To assess whether the protocol established for Gram-negative bacteria was easy-fitting to Gram-positive cocci as well, we performed preliminary experiments. Since the results obtained were not as satisfactory as those obtained with Gram-negative bacteria, the protocol was slightly modified. Addition of a protein extraction step (see materials and methods) to the bacteria placed onto the polished steel target plate of MALDI-TOF allowed obtaining favourable results by the direct method. Therefore, we applied this modification of the direct method to all the cultures containing Gram-positive cocci. For comparison, we decided to apply in parallel on the same sample a commercially available kit, the SepsiTyper, which has been released to facilitate the purification and extraction of the bacterial proteome from positive blood cultures. The results obtained by the direct and the SepsiTyper methods were compared with those by the routine methods used in our laboratory. The identification of Gram-positive cocci from positive blood cultures by MALDI-TOF with the direct and the SepsiTyper method gave concordant results with routine methods in 72 (90 %) and 70 (87 %) of the 80 monomicrobial blood cultures, respectively. Statistical analyses revealed no significant differences in the identification ability between the direct and the SepsiTyper method by MALDI-TOF. The scores were as follows (Table 3): 2 (2 %) and 7 (8 %) score ≥ 2.3, 24 (30 %) and 23 (29 %) score between 2.3 and 2.0, 26 (33 %) and 29 (36 %) score between 2.0 and 1.7, and 20 (25 %) and 11 (14 %) score < 1.7. Overall, 26 (32 %) and 30 (37 %) of 80 blood cultures showed scores > 2.0. Five (6 %) and 6 (8 %) isolates processed by the direct and the SepsiTyper method, respectively, were unidentified by MALDI-TOF, due to the absence of peaks. In these cases, the bacteria spotted in duplicate gave different bacterial identification results. By the direct method, the unidentified strains were 3 *Staphylococcus epidermidis*, 1 *Staphylococcus haemolyticus*, and 1 *Micrococcus luteus*, and by the SepsiTyper method were 1 *Staphylococcus aureus*, 1 *S. epidermidis*, 1 *Staphylococcus capitis*, 1 *S. haemolyticus*, 1 *Enterococcus faecium*, and 1 *M. luteus*. The misidentified strains were 3 (4 %) and 4 (5 %) by the direct and the SepsiTyper method, respectively. The 3 misidentified strains by the direct method were: 1 *S. haemolyticus* erroneously identified as *Staphylococcus hominis*, 1 *E. faecium* as *Enterococcus faecalis*, and 1 *Streptococcus oralis* group mitis as *Streptococcus pneumoniae*. The 4 misidentified strains by the SepsiTyper method were 1 *S. epidermidis*, erroneously identified as *S. aureus*, 1 *Staphylococcus warneri* as *Staphylococcus pasteuri*, 1 *E. faecium* as *E. faecalis*, and 1 *S. oralis* group mitis as *S. pneumoniae*. Therefore, the SepsiTyper method did not discriminate coagulase-negative staphylococci from *S. aureus* in 1 out of 68 samples.

**Table 3:** Identification of Gram-positive cocci from monomicrobial positive blood cultures by MALDI-TOF (direct and SepsiTyper (ST) methods)*

	No. of isolates												
Species	Identification by use of MALDI-TOF MS with score (S) of:								No. of unidentified isolates		No. of misdentified isolates		Total (%)
	S < 1.7		1.7 ≤ S < 2		2.0 ≥ S <2.3		S ≥ 2.3						
	Direct	ST	Direct	ST	Direct	ST	Direct	ST	Direct	ST	Direct	ST	
*S. aureus*	1		3		2	4		1		1			6 (7.5 %)
*S. epidermidis*	14	11	16	20	3	3			3	1		1	36 (45 %)
*S. capitis*			1	1	5	5	1			1			7 (8.9 %)
*S. haemolyticus*	1		1	2	3	3		1	1	1	1		7 (8.9 %)
*S. hominis*	2			1	4	3		2					6 (7.5 %)
*S. warneri*	1		1	1								1	2 (2.5 %)
*Staphylococcus xylosis*			1	1									1 (1.2 %)
*Staphylococcus simulans*					1	1							1 (1.2 %)
*Staphylococcus sciuri*					1	1							1 (1.2 %)
*Staphylococcus pettenkoferi*			1	1									1 (1.2 %)
*E. faecium*				1	4	1		1		1	1	1	5 (6.3 %)
*E. faecalis*					1	1	1	1					2 (2.5 %)
*Enterococcus casseliflavus*			1			1							1 (1.2 %)
*S. oralis* group mitis											1	1	1 (1.2 %)
*Streptococcus anginosus*			1	1									1 (1.2 %)
*M. luteus*	1							1	1	1			2 (2.5 %)
Total (%)	20 (25 %)	11 (14 %)	26 (33 %)	29 (36 %)	24 (30 %)	23 (29 %)	2 (2 %)	7 (8 %)	5 (6 %)	6 (8 %)	3 (4 %)	4 (5 %)	80

*Using the results obtained with the routine methods by MALDI-TOF and Vitek 2 as comparators

Among the 7 positive blood cultures that resulted to be polymicrobial after subculture, one microorganism of the mixture was correctly identified to the species level in all (100 %) samples by the direct method, and in 6 (86 %) out of 7 by the SepsiTyper method (Table 4). MALDI-TOF analyses of bacteria spotted in duplicate gave 100 % concordance and similar scores. In 2 cases both species were identified: one by the direct and the other by the SepsiTyper method. The MALDI-TOF identification scores are reported in Table 4. No incorrect results were given. The mean times required for the identification of Gram-positive bacteria from positive blood cultures was 25 min for the direct method and 20 min for the SepsiTyper method.

**Table 4:** Identification by MALDI-TOF (direct and SepsiTyper (ST) methods) of Gram-positive cocci from positive blood cultures appearing monomicrobial at the Gram-stain, that resulted to be polymicrobial after subculture*

	No. of isolates											
Species	Identification by use of MALDI-TOF MS with score (S) of:								No. of unidentified isolates		Unidentified second species	Total samples
	S < 1.7		1.7 ≤ S < 2		2.0 ≥ S <2.3		S ≥ 2.3					
	Direct	ST	Direct	ST	Direct	ST	Direct	ST	Direct	ST		
*S. aureus*			1					1			*S. capitis*	
*S. epidermidis*	1			1							*E. faecalis*	
*S. capitis*					1	1^a^					*E. faecium*	
*S. capitis*			1	1^a^							*S. epidermidis*	
*S. hominis*					1					1	*S. haemolyticus*	
*S. hominis*					1			1			*S. epidermidis*	
*E. faecium*				1			1				*S. epidermidis*	
Total (%)	1 (14 %)		2 (29 %)	3 (43 %)	3 (43 %)	1 (14 %)	1 (14 %)	2 (29 %)		1 (14 %)		7

*Using the results obtained with the routine methods by MALDI-TOF and Vitek 2 as comparators

^a^ST correctly identified only the second species

## Discussion

MALDI-TOF MS technology has rapidly evolved during the last few years, making it possible species identification of both gram-positive and gram-negative bacteria in a few minutes. Although originally devised for identification of bacteria from isolated colonies, this methodology has been recently adapted and successfully used for identification of bacteria directly from positive blood cultures without the need of subculture onto solid medium [12, 16–19, 21, 22]. The main conclusion from the present study is that the deposition of Gram-negative or Gram-positive bacteria harvested by SST from a positive blood culture bottle into the steel target plate can be used for rapid and reliable identification by MALDI-TOF. Our conclusion is based on the following findings. First, concordant identification of the monomicrobial Gram-negative bacteria by the direct MALDI-TOF identification method compared to the routine methods by MALDI-TOF and Vitek 2 was 100 % and 99.2 %, respectively. The spectra from blood cultures most often showed high confidence identification scores, though in a few cases spectra from blood cultures were of lower quality than those from colonies. However, this problem, which has been previously noticed also by other authors (14), did not compromise the results in this instance. Consistently with other authors, species belonging to *Enterobacter cloacae* group could not be separated definitively by the direct method, due to the few differences in the proteomic profiles within the group [17, 19, 20]. Therefore, the identification results for *Enterobacter* spp. were performed by MALDI-TOF on isolated colonies and reported to the clinician when the first four best matches indicated the same species. One isolate that was identified as *S. maltophilia* by Vitek 2, was not identified either by the direct and routine methods by MALDI-TOF.

Second, concordant identification of monomicrobial Gram-positive cocci by MALDI-TOF with the direct and the SepsiTyper method compared to the routine methods was 90 % and 87 %, respectively, despite the low spectral scores. Like other authors, who have reported on the use of MALDI-TOF directly from positive blood cultures, we observed that cutoff values could be lowered down to 1.4 without compromising accuracy [21, 23, 24]. Strains were unidentified by the direct and the SepsiTyper method in 6 % and 8 %, and misidentified in 4 % and 5 %, respectively. As at present MALDI-TOF technology is not able to readily distinguish between *S. oralis* group mitis and *S. pneumoniae*, it seems prudent to report isolates that meet identification criteria as *S. pneumoniae*/*S. mitis* group pending additional identification tests [17, 25]. In fact, several other authors also experienced such a limitation with streptococci by using different MALDI-TOF systems and sample preparation protocols [18, 24, 26, 27].

Among the apparently monomicrobial samples that resulted to be polymicrobial for Gram-negative bacteria or Gram-positive cocci after subculture, one of the Gram-negative bacilli or Gram-positive cocci of the mixture was identified in 87 % and 100 % of the samples. In 2 (29 %) out of 7 samples polymicrobial for Gram-positive cocci, both the Gram-positive cocci were correctly identified, one by the direct and the other by the SepsiTyper method. Yeasts, that were not visible at the Gram-stain, were not identified in polymicrobial samples. Differently from other authors [12], we experienced no false positive results either in monomicrobial or polymicrobial samples.

Lastly, the direct method allows identification of bacteria in less than 30 min following blood culture positivity. Although the time required for identification by the SepsiTyper was similar, the latter method involves several centrifugation steps, longer processing time and additional costs. Unlike other protocols requiring an additional incubation of positive blood culture samples in liquid or solid medium [28, 15], the method proposed in this study avoids further incubation steps. Another newly developed method is lysis filtration, by which rapid and reliable bacterial identification results have been obtained, though important microorganisms like *K. pneumoniae* and *S. aureus* were not identified in 1 case each, possibly due to cell debris remaining on the membrane [16]. This reminds us that sample processing is a critical step for correct identification by MALDI-TOF and even subtle differences in sample preparation may influence the final results.

Further studies analysing a higher number of samples would allow a more precise evaluation of the accuracy of the different methods that were comparatively analysed in the present study. Moreover, MALDI-TOF MS currently does not provide adequate data on antimicrobial susceptibility. Hence, there will be a continuing requirement for bacterial culture.

## Conclusions

In conclusion, in this study we propose the application of a simple method for MALDI-TOF identification, using bacteria harvested from blood cultures by SST and placed onto the polished steel target plate. Addition of a protein extraction step directly onto the steel target plate resulted to be essential for correct identification of Gram-positive cocci. The developed method is rapid (less than 30 min) and reliable, not requiring particularly skilled personnel or the use of expensive commercially available kits. Concordant results between the direct and routine method by MALDI-TOF were found for 100 % Gram-negative bacteria (96 % with score > 2.0) and 90 % Gram-positive cocci (32 % with score > 2.0). For comparison, SepsiTyper gave concordant identification results of Gram-positive cocci with routine identification by MALDI-TOF for 87 % of cases (37 % with score > 2.0). In addition, the incorrect results obtained with the direct method on Gram-positive cocci would not result in very different clinical outcomes. The good agreement between direct MALDI-TOF identification and routine methods suggests that the direct MALDI-TOF identification method can be used to report reliable results and streamline empirical antimicrobial therapy in patients with bloodstream infections a day earlier than the current method.

## Materials and methods

### Blood samples

Blood specimens, from patients admitted to Pisa University Hospital (Italy) in the period January-July 2013, were inoculated into blood culture bottles [Plus Aerobic/F and Plus Anaerobic/F, or Peds Plus F (Becton Dickinson & Co, BD, Milan, Italy)], collected at the Unità Operativa di Microbiologia Universitaria, and transferred to the Bactec FX instrument (Becton Dickinson, Franklin Lakes, NJ, USA) for monitoring the bacterial growth. From each patient, only the first positive blood culture apparently monomicrobial at the Gram-stain was included in this study. Blood cultures containing Gram-negative bacilli from 133 patients or Gram-positive cocci from 87 patients were investigated. After subculture on blood agar plates (BD), 15 (11 %) of 133 and 7 (8 %) of 87 cultures were found to be polymicrobial and analyzed separately.

The study was notified to the local committee, Comitato Etico di Area Vasta Nord-Ovest, University of Pisa, and conducted in full accordance with the principles of the Declaration of Helsinki. Samples were taken as part of the standard patient care and used anonymously. For this type of study no written informed consent was necessary.

### Routine methods of blood culture processing and identification of microorganisms

Positive blood culture bottles at the Bactec FX were Gram-stained, subcultured, and incubated overnight at 37 °C. Routine identification of isolated colonies was performed by the Vitek 2 system (Vitek 2 software, version 05.04; Advanced Expert System software, version 1.9.0; bioMérieux, Marcy l’Étoile, France). In parallel, bacteria grown on solid media were spotted onto the polished steel target plate for MALDI-TOF (Bruker Daltonics, Bremen, Germany) identification.

### Sample preparation for the identification of bacteria by the direct method

An eight-ml sample of a positive blood culture bottle at the Bactec FX was transferred to Serum Separator Tubes (BD Vacutainer systems). Next, bacteria were sedimented on the surface of the silicon layer by centrifugation at 2,000 × g for 10 min. A suspension of Gram-negative bacilli (0.8 McFarland) or Gram-positive cocci (3–3.5 McFarland) was prepared in 1 ml of distilled water and transferred into an eppendorf tube. After centrifugation at 13,000 rpm for 2 min, the bacterial pellet was allowed to dry at room temperature. Next, bacteria were transferred by a micropipette tip onto the polished steel target plate for MALDI-TOF identification. Gram-positive cocci were further exposed to a protein extraction protocol (see below).

### Protein extraction protocol on the steel target plate

Gram-positive cocci placed onto the steel target plate were overlaid with 0.6 μl absolute ethanol (Fluka, St. Louis, MO, USA). When dry, 0.6 μl formic acid (70 % v/v; Fluka) was added and, when dry, an equal volume (0.6 μl) of acetonitrile (Carlo Erba, Milan, Italy) was added. When dry, the preparation was overlaid with the matrix solution for MALDI-TOF identification (see below).

### Sample preparation for the identification of Gram-positive cocci by the SepsiTyper method

Sample preparation using the SepsiTyper method (Bruker Daltonics, Bremen, Germany) was performed according to the manufacturer’s instructions. Briefly, 1 ml of a positive blood culture was transferred to an eppendorf tube and vortexed upon addition of the lysis buffer (200 μl). Samples were centrifuged at 13,000 rpm for 2 min. The pellet was suspended in 1 ml washing buffer and centrifuged at 13,000 rpm for 1 min. Next, the pellet was suspended in 300 μl of deionized water. Then, absolute ethanol (900 μl) was added to the suspension. The mixture was centrifuged at 13,000 rpm for 2 min. Next, 50 μl formic acid (70 % v/v) was added to the pellet followed by the addition of 50 μl acetonitrile. The mixture was centrifuged at 13,000 rpm for 2 min and 1 μl of the supernatant was spotted onto the polished steel target plate for MALDI-TOF identification.

### Mass spectrometry identification and analysis

The collected bacteria were spotted in duplicate onto a polished steel target plate and when dry, the preparation was overlaid with 1 μl of saturated alpha-cyano-4-hydroxycinnamic acid in 50 % acetonitrile and 2.5 % trifluoroacetic acid matrix solution (HCCA Bruker Daltonics) and air dried, thus allowing to cocrystallize with the sample before further processing with MALDI-TOF.

MALDI-TOF analysis was performed using a Microflex LT system table top mass spectrometer following the manufacturer’s instruction settings. Captured spectra were analysed using a MALDI-TOF Biotyper automated control and the Bruker Biotyper 3.1 software and library (4624 isolates) (Bruker Daltonics). For each plate, bacterial test standard (Bruker Daltonics) dissolved in organic solvent (consisting of 50 % acetonitrile and 2.5 % trifluoroacetic acid) was included to calibrate the instrument and validate the run.

Criteria used for analysis were as recommended by the manufacturer for colony identification. Briefly, scores were interpreted as follows: < 1.7 as unreliable identification, 1.7-1.999 as valid identification to the genus level, 2.0-2.299 as definite identification of the genus and probable identification of the species, and ≥ 2.300 as certain identification of the genus and high probability of species identification. Therefore, valid identifications were those with a score of ≥ 2.0.

### Data analysis

Identification data obtained by the direct method were evaluated using the data from the routine methods by MALDI-TOF and Vitek 2, as comparators. The identification results were classified as concordantly identified, misidentified (where the bacterium was incorrectly identified at the genus or species level), and unidentified. Discrepancies in identification of the bacteria between the direct and the routine methods were resolved with complementary biochemical tests.

Statistical analyses was performed using the chi-square test for independent pairs with Yates’ correction if necessary. A *P* value of < 0.05 was considered statistically significant.
